# A novel multi-level 3D pose estimation framework for gait detection of Parkinson’s disease using monocular video

**DOI:** 10.3389/fbioe.2024.1520831

**Published:** 2024-12-23

**Authors:** Rong He, Zijing You, Yongqiang Zhou, Guilan Chen, Yanan Diao, Xiantai Jiang, Yunkun Ning, Guoru Zhao, Ying Liu

**Affiliations:** ^1^ Department of Rehabilitation Medicine, University of Hong Kong-Shenzhen Hospital, Shenzhen, China; ^2^ CAS Key Laboratory of Human-Machine Intelligence-Synergy Systems, Research Center for Neural Engineering, Shenzhen Institutes of Advanced Technology, Chinese Academy of Sciences, Shenzhen, China; ^3^ University of Chinese Academy of Sciences, Beijing, China

**Keywords:** Parkinson’s disease (PD), 3D pose estimation, monocular video, graph convolutional network (GCN), gait detection

## Abstract

**Introduction:**

Parkinson's disease (PD) is characterized by muscle stiffness, bradykinesia, and balance disorders, significantly impairing the quality of life for affected patients. While motion pose estimation and gait analysis can aid in early diagnosis and timely intervention, clinical practice currently lacks objective and accurate tools for gait analysis.

**Methods:**

This study proposes a multi-level 3D pose estimation framework for PD patients, integrating monocular video with Transformer and Graph Convolutional Network (GCN) techniques. Gait temporal and spatial parameters were extracted and verified for 59 healthy elderly and PD patients, and an early prediction model for PD patients was established.

**Results:**

The repeatability of the gait parameters showed strong consistency, with most of the estimated parameters yielding an Intraclass Correlation Coefficient (ICC) greater than 0.70. Furthermore, these parameters exhibited a high correlation with VICON and ATMI results (*r* > 0.80). The classification model based on the extracted parameter features, using a Random Forest (RF) classifier, achieved an accuracy of 93.3%.

**Conclusion:**

The proposed 3D pose estimation method demonstrates high reliability and effectiveness in providing accurate 3D human pose parameters, with strong potential for early prediction of PD.

**Significance:**

This markerless method offers significant advantages in terms of low cost, portability, and ease of use, positioning it as a promising tool for monitoring and screening PD patients in clinical settings.

## 1 Introduction

Parkinson’s disease (PD) is a chronic progressive neurodegenerative disease, mainly caused by the degeneration and death of dopaminergic neurons in the substantia nigra. It is related to multiple factors such as genetics, environment, and aging of the nervous system (Bloem et al., 2021). Dopamine, a critical neurotransmitter, plays a pivotal role in regulating voluntary movement in the human body. Dopamine deficiency usually provokes symptoms of significant gait disturbance (Armstrong and Okun, 2020) Currently, the prevalence of PD in the elderly over 65 years old in China is 1.7%, and the number of PD patients exceeds 3 million (Qi et al., 2021). PD is typically manifested by body tremors (usually starting in the hands), muscle stiffness, bradykinesia, and balance disorders, and may also lead to loss of smell, constipation, sleep disorders, etc. (Poewe et al., 2017). Gait motor disorder is among the most prevalent motor impairments in PD, and gait analysis is closely linked to the assessment of disease severity (Morris and Iansek, 1996a). Statistics indicate that over 60% of patients with late-stage Parkinson’s disease (PD) develop severe gait disorders, including freezing of gait (Virmani et al., 2015). Most patients in late-stage are unable to walk independently. At the same time, those with milder symptoms exhibit gait patterns that are often challenging to differentiate from those of healthy individuals, necessitating careful observation and assessment by clinicians. Therefore, research on posture estimation and gait analysis in patients with milder Parkinson’s offers a pathway to rapid and objective early diagnosis. Providing timely interventions based on these analyses is anticipated to delay disease progression and enhance patients’ quality of life (Di Biase et al., 2020).

Currently, the diagnosis and evaluation of PD mainly include clinical scales and commercial gait analysis equipment. Clinical scales include the PD Rating Scale (UPDRS), gait evaluation scales (Hoehn-Yahr Scale, MDS-UPDRS-III Scale, Berg), etc., which mainly rely on doctor experience and are subjective (Di Biase et al., 2020). Commercial equipment includes the marker motion capture system VICON, the wearable gait analysis system XSENS, and the three-dimensional plantar pressure monitoring system ATMI, which can provide high-precision, objective, and effective results, but are expensive and complicated to operate (Merriaux et al., 2017; Parati et al., 2022; Benjaminse et al., 2020). In addition, gait analysis methods based on inertial wearable measurement units (IMUs) have been widely used in recent years for motion monitoring of patients with neuro-motor degenerative diseases. Gu et al. developed a non-contact real-time muscle activity measurement system that uses two IMUs to capture the motion data during walking and is used to evaluate the flexion and extension of the knee (Gu et al., 2022). Mallat et al. proposed a new portable and easy-to-operate visual-inertial motion capture system that integrates IMU data with visual information into a biomechanical model to improve the accuracy of pose estimation (Mallat et al., 2022). Nevertheless, IMU performs poorly in estimating pose angles and suffers from signal drift problems, so new clinical tools need to be developed to meet the needs of PD gait monitoring.

Markerless human pose estimation technology can analyze video data based on deep learning and image processing methods and has become a new method for motion capture, with the advantages of portability and simplicity (Altham et al., 2024). At present, clinical studies have used single or multiple cameras to capture patient motion data and assess the severity of patients (Hulleck et al., 2022). Li et al. conducted a visual-based pose estimation assessment study on PD and levodopa-induced dyskinesia (LID), and successfully extracted features related to PD and LID diseases (Li et al., 2018). Guo et al. proposed an automated five-category freezing of gait (FoG) assessment method based on graph convolutional networks (GCN), using multi-camera motion video training data to achieve a classification accuracy of 91.32%, providing a new solution for PD diagnosis and management (Guo et al., 2024). Zheng et al. proposed a video analysis method based on a skeleton-contour fusion convolutional network to predict the MDS-UPDRS gait score of PD patients. This method utilized dual-view imaging, incorporating sagittal and coronal perspectives, and achieved a prediction accuracy of 71.25% on 80 early patients and healthy subjects (Zeng et al., 2023). Yuki et al. proposed a video-based FoG automatic detection algorithm that combines target tracking and 3D pose estimation. Utilizing multi-view video recordings from routine clinical practice involving PD patients, the method demonstrated a remarkable accuracy of 93.2% in distinguishing FoG from walking stops (Kondo et al., 2024). However, current research tends to use multi-camera data acquisition, which significantly increases the complexity of the system and data processing. The acquisition method of a monocular camera is more convenient and cheaper, which has the potential to be more suitable for clinical promotion.

Markerless human pose estimation mainly includes 2D and 3D methods. Among them, 2D pose estimation focuses on the position of human joints in the two-dimensional plane, while 3D pose estimation focuses on the precise coordinates of human joints in three-dimensional space, which is more suitable for motion capture and trajectory analysis of PD patients (Chen et al., 2020). Tang et al. proposed a Transformer-based 3D human pose estimation model STC-Former, which combined the STC and SPE modules to improve the recognition accuracy of the spatiotemporal relationship and local structure of human joints (Tang et al., 2023). Yu et al. proposed a GLA-GCN model that combined global (Bloem et al., 2021) spatiotemporal and local joint representations and introduced individual connection layers for 3D pose estimation, achieving 3% and 17% error reduction on the Human3.6 M and HumanEva-I datasets, respectively (Yu et al., 2023). Tang et al. proposed a 3D human pose estimation method MH-Former based on Transformer learning multiple hypothesized spatial and temporal representations, which effectively solved the challenges of depth blur and self-occlusion in monocular video 3D pose estimation (Li et al., 2021). Li et al. integrated human skeletal structure information and prediction uncertainty designed a new self-attention mechanism and position encoding, and adopted sampling and refinement strategies to significantly improve the accuracy of 2D to 3D human pose estimation (Li H. et al., 2023). Hu et al. developed a high-resolution graph convolutional multi-layer perception (HGcnMLP) algorithm, which achieved 3D human pose estimation of patients with musculoskeletal diseases through smartphone monocular video, which was highly consistent with the VICON system (Hu et al., 2024). Gholami et al. proposed an automatic gait labeling method for PD based on weakly supervised learning, with an accuracy of 89% in data detection of PD patients (Gholami et al., 2023). Although the current research on unlabeled 3D pose estimation methods for monocular videos has made great progress, there are still problems such as low accuracy and poor robustness that limit its practical application in clinical evaluation of PD patients.

Therefore, to improve the accuracy and stability of the model and realize the evaluation of clinical gait motion function, this work intends to develop a 3D pose estimation method for PD patients based on monocular video and verify the feasibility of early diagnosis. The main objectives include: 1) Construct a multi-level 3D pose feature extraction framework based on Transformer and GCN networks, which can analyze motion data from the perspectives of time series and spatial structure respectively; 2) Extract gait temporal parameters and spatial parameters of PD patients based on monocular video, and verify their repeatability and reliability; 3) Construct a prediction model for early PD patients and healthy elderly people to achieve accurate prediction of patients.

## 2 Experiment

**FIGURE 1:**
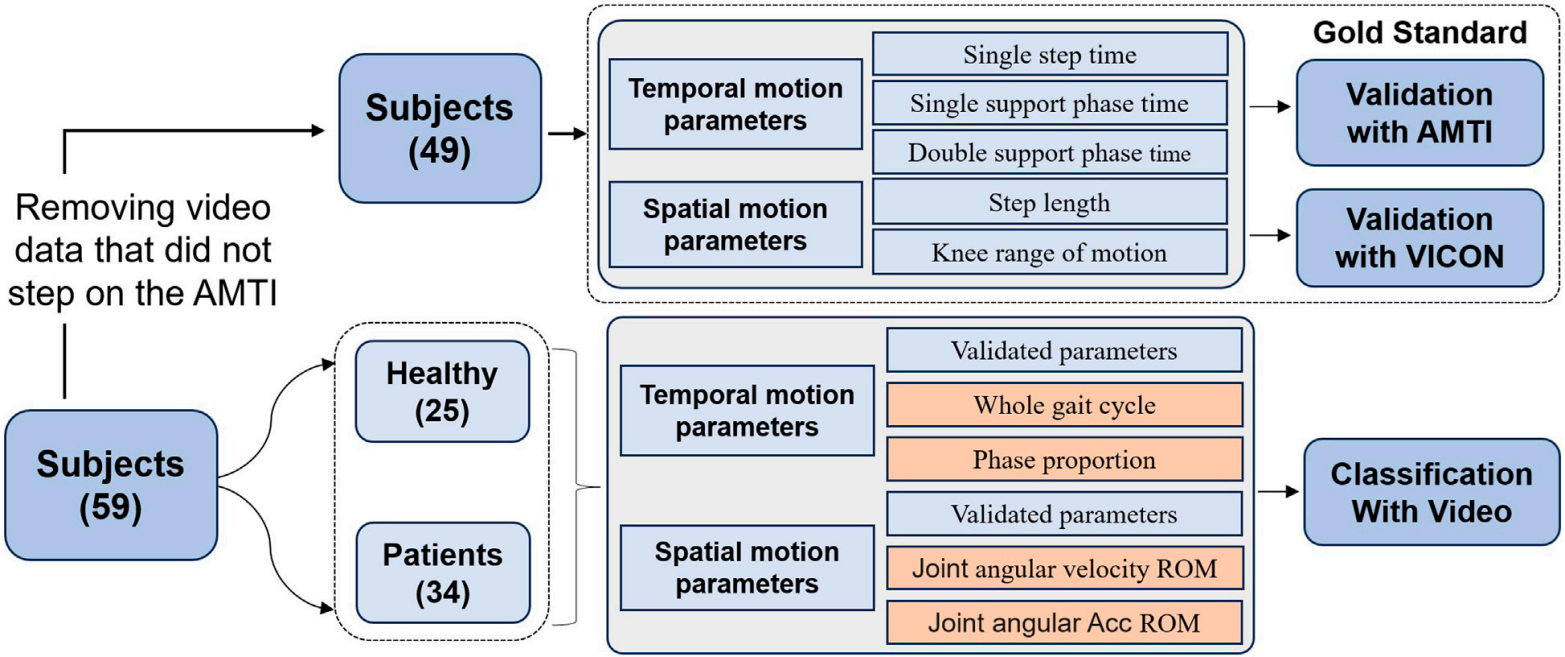
The flowchart and objectives of this research.

The comprehensive experimental workflow of this study is illustrated in Figure 1. The experimental validation phase focused on two systems: the VICON optical motion capture system and the plantar 3D pressure measurement system. The VICON system captured kinematic parameters and spatio-temporal parameters, such as step length and joint angles. The plantar pressure measurement system simultaneously collects kinetic characteristics and completes the segmentation of gait phases. The collected data can be used to assess the reliability and validity of the proposed algorithm framework. The classification phase analyzes the dataset extracted from video information to identify and differentiate healthy individuals from patients by leveraging characteristics that exhibit significant differences.

### 2.1 Participants

A total of 59 participants were recruited for this study, including 25 healthy elderly and 34 PD patients. All subjects were recruited from the University of Hong Kong Shenzhen Hospital. The criteria for PD participant selection are as follows: 1) Patient is clinically diagnosed with Parkinson’s disease; 2) Patients have bradykinesia, along with either resting tremor or rigidity; 3) Patients have no diseases other than PD that impair walking ability and can walk independently. 4) Patients exhibit no cognitive impairment, have no history of diabetes, and can complete all tests independently. Table 1 shows the demographic characteristics of the participants. Before the experiment, a professional physical therapist assessed the severity of Parkinson’s disease in participating patients using the H-Y clinical scale and gait tests. All participants signed an informed consent form, and all relevant experiments involving humans were approved by the Medical Research Ethics Committee of the University of Hong Kong Shenzhen Hospital (Ethics No. hkuszh2023089).

**TABLE 1:** Demographic characteristics of patients and healthy elderly.

Characteristics	Mean ± Standard Deviation		
	Healthy adults	Patients (mild + medium)	
Number	25	19	15
Disease duration	0	6.05 ± 4.55	6.80 ± 5.90
Age (years)	55.36 ± 5.77	69.79 ± 10.66	62.67 ± 10.61
Weight (kg)	62.92 ± 10.41	60.39 ± 10.33	61.53 ± 6.46
Height (cm)	158.08 ± 8.20	161.74 ± 7.76	161.47 ± 5.83
H-Y	0	1.34 ± 0.76	1.87 ± 0.79

### 2.2 Experimental setup

**FIGURE 2:**
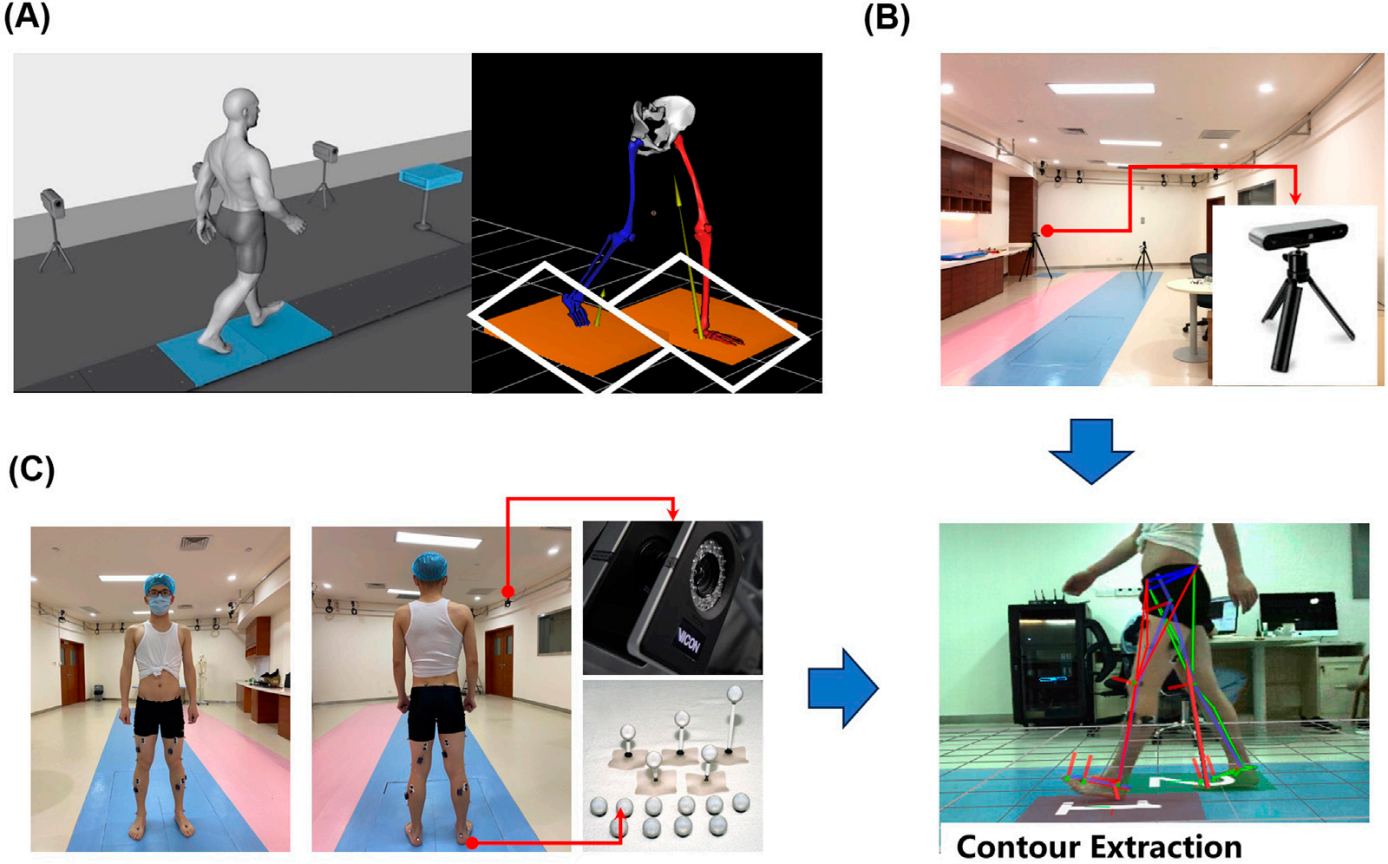
Experimental setup, **(A)** 3D plantar pressure measurement system setup, **(B)** depth camera position schematic, **(C)** VICON experimental configuration.

The VICON motion capture system (Vicon Ltd., Oxford, United Kingdom) was used, consisting of 12 MX infrared microphotographic cameras to capture lower limb kinematic data with a sampling frequency of 100 Hz; two force plates (AMTI, Optima HPS, United States) with a sampling frequency of 1,000 Hz, were used to synchronously collect mechanical signals acting on the platform, including three-dimensional plantar pressure information, the center of pressure (COP), and various kinetic parameters calculated by the analysis software. The AMTI force plates and VICON system hardware devices were connected to the same synchronized signal source. A monocular camera was utilized to capture motion videos. The experimental setup is depicted in Figure 2.

(a) In a laboratory with an area of 15 m × 8 m, an effective capture space region of 1.8 m × 0.8 m was established. Two force measurement plates, each measuring 0.6 m × 0.405 m, were embedded into a 3 m long wooden walkway (Figure 2A). (b) Video data was collected by a monocular camera positioned perpendicular to the forward direction of the human body, located 2.8 m from the force plate runway (Figure 2B). The camera has a resolution of 1,280 × 720 pixels at a frame rate of 30 Hz (fixed-focus mode). (c) Twelve motion capture cameras (MoCap) were placed on a wall frame, markers were positioned according to the 16 reflective marker points plug-in model recommended by Visual 3D (C-motion Inc., Kingston, Canada), and 16 marker balls were attached to the bony prominences of the lower extremities bilaterally by a professional physiotherapist (Figure 2C).

### 2.3 Data acquisition

Before each test, participants performed the task in the following order: standing-walking-stopping-turning-walking-stopping. The walking distance for a single trial was 8 m, with each movement sequence repeated three times. The testing interval lasted until the patient felt tired, usually more than 2 min. During the data collection, two walking cycles were selected for each subject while walking close to the range of the force plate. Throughout these cycles, the dynamic joint angles, gait spatial-temporal parameters, and plantar pressure parameters were observed. All operations were carried out with professional physiotherapists.

## 3 Methods

### 3.1 Data preprocessing

A rigorous data preprocessing protocol was implemented for the video data. In the validation experiment, synchronization criteria were applied: only gait segments synchronized with both the Vicon motion capture system and AMTI force platform measurements were retained, resulting in a final dataset comprising 49 participants with corresponding segments. For the reliability analysis, a robust quality control framework was implemented. Frames with incomplete participant visibility were systematically excluded, two gait cycles within the force plate range were retained for each video clip, and only participants with at least three valid video segments were included in the analysis. To ensure data consistency, three segments per participant were extracted, yielding a standardized dataset of 177 segments from 59 participants. In the subsequent variability and classification experiments, gait features were extracted from each of the three segments per participant from the reliability study dataset, and their mean values were computed, generating 59 feature sets for comprehensive analysis.

### 3.2 Human 3D poses estimation framework

#### 3.2.1 Extracting 2D key points

**FIGURE 3:**
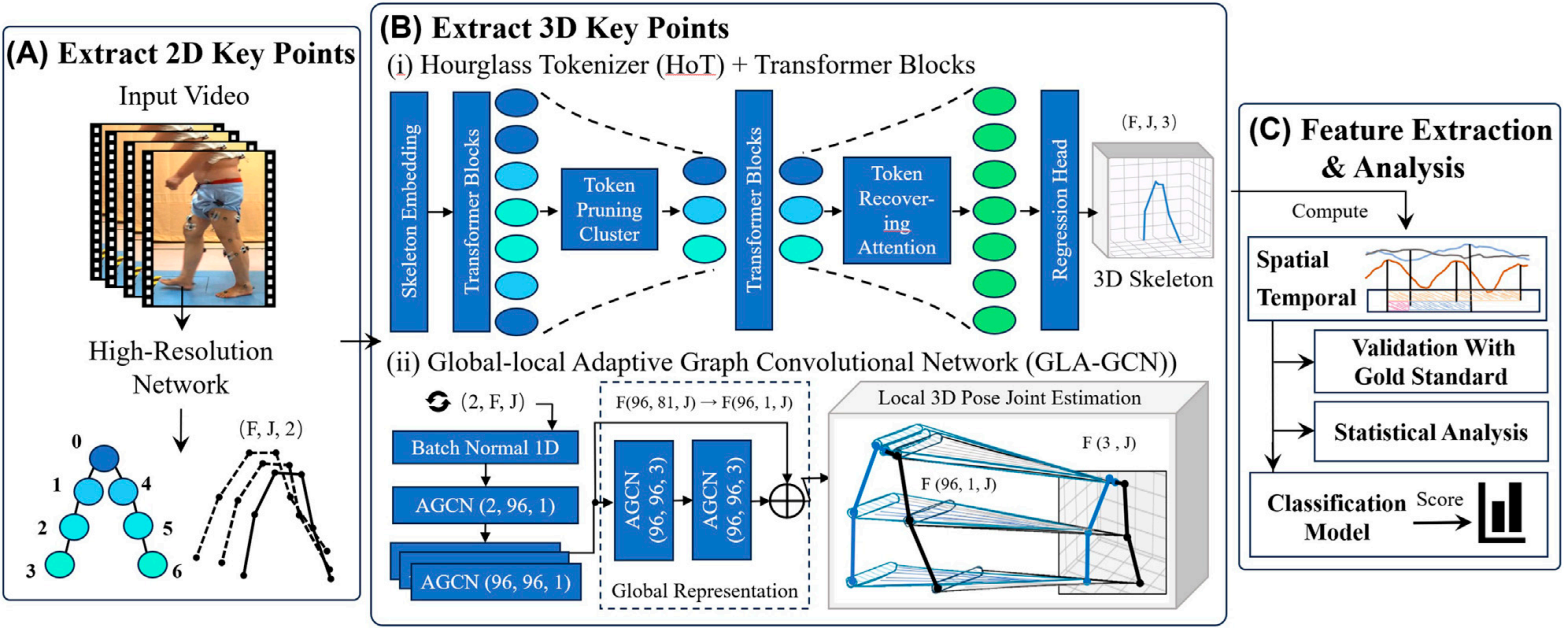
The flowchart of the method includes extracting 2D key points, Extracting 3D key points, feature extraction and data analysis. **(A)** Extract 2D key points. **(B)** Extract 3D key points **(C)** Feature extraction and analysis (i) Hourglass Tokenizer (HoT) Transformer Blocks. (ii) Global-local Adaptive Graph Convolutional Network (GLA-GCN).

Cheng et al. proposed a high-resolution network (HRNet) that can maintain high resolution throughout the propagation process and perform repeated multi-scale fusion and is widely used as a 2D input model in 3D key point detection (Cheng et al., 2019). In 2D key point extraction, the width, height, frame rate, and other information of video data are obtained based on the OpenCV library (Bradski, 2000), and object detection is performed at the initial frame of the video clip to ensure the presence of a human body in the extracted frames (Bradski, 2000). Subsequently, the HRNet model was used to output 2D key points for human pose estimation (Figure 3A).

#### 3.2.2 Extracting 3D key points

##### 3.2.2.1 Hourglass tokenizer (HoT)

The HoT framework (Li et al., 2023b) was employed to efficiently leverage the sequence-to-sequence (seq2seq) approach within Video Pose Transformers (VPTs) for 3D human pose estimation (Figure 3B (i)). Through seq2seq, a 2D pose sequence for each frame $x_{n}\in R^{F\times J\times C}$ , where J and F are the number of body joints, and input frames and C denotes the feature dimension, is input to output a 3D pose sequence across all frames (Zhang et al., 2022). HoT is a plug-and-play framework that utilizes the Token Pruning Cluster (TPC) and Token Recovering Attention (TRA) modules to efficiently prune and recover tokens. TPC dynamically captures pose tokens representing keyframes. For the input pose tokens at the n−th Transformer block, average spatial pooling is applied along the spatial dimension to mitigate spatial redundancy. A density-peak clustering algorithm based on k-nearest neighbors (DPC-kNN) is then applied to identify the most representative tokens by maximizing intra-class proximity and inter-class distance, discarding redundant tokens across the time dimension.

For a given token $x^{i}\in {\bar{x}}_{n}$ , the local density of tokens ρ is computed as Formula 1:

$$\rho_{i}=\exp\left(-\frac{1}{k}\sum_{x^{j}\in kNN\left(x^{i}\right)}{\left\|x^{i}-x^{j}\right\|}_{2}^{2}\right)$$ (1)

Where $kNN\left(x^{i}\right)$ represents the k-nearest neighbors of token $x^{i}$ .

The clustering center score $\delta_{i}$ is defined as Formula 2:

$$\delta_{i}=\left\{\begin{array}{l} \min_{j:\rho_{j}>\rho_{i}}{\left\|x^{i}-x^{j}\right\|}_{2},if\exists \rho_{j}>\rho_{i}\\ \max_{j}{\left\|x^{i}-x^{j}\right\|}_{2},otherwise \end{array}\right.$$ (2)

To restore the temporal resolution of the original video, the TRA module leverages a multi-head cross-attention (MCA) mechanism. Using a lightweight network, it recovers the pruned spatiotemporal information. The recovered tokens are then passed to the regression head, which outputs a 3D pose sequence $q\in R^{F\times J\times 3}$ , achieving 3D pose estimation for each frame.

##### 3.2.2.2 Global-local adaptive graph convolutional network (GLA-GCN)

The Global-Local Adaptive Graph Convolutional Network (GLA-GCN) (Yu et al., 2023) is utilized, which extracts human pose structure information through adaptive graph convolution of global spatiotemporal representations, combined with local fine-grained estimation (Figure 3B (ii)). In the global representation, an Adaptive Graph Convolutional Network (AGCN) constructs a global spatiotemporal graph from the input 2D pose sequence, leveraging graph convolution to extract both spatial and temporal pose features. Joints are classified into three categories: the vertex itself, the centripetal subset (joints closer to the body’s center of mass), and the centrifugal subset (joints farther from the center).

The local 3D pose joint estimation is carried out using two independent fully connected layers: one with non-shared weights and the other with shared weights. The non-shared layer assigns unique weights to each joint, while the shared layer applies the same weights across all joints. The final 3D position of each joint is estimated via a weighted average of these two layers.

The individual connected layer can be denoted as Formula 3:

$${\dot{p}}_{i}^{\left(unshared\right)}=v_{i}W_{i}+b_{i}$$ (3)

Here, ${\dot{p}}_{i}$ represents the 3D position estimate of joint i , and $v_{i}$ denotes the flattened feature of joint i . The weight of the single fully connected layer is denoted as Wi , $b_{i}$ representing the bias term.

The shared fully connected layer is expressed as Formula 4:

$${\dot{p}}_{i}^{\left(shared\right)}=v_{i}W_{s}+b_{s}$$ (4)

Where Ws denotes the weight parameters of the shared layer, and $b_{s}$ represents the bias term.

The weighted average is computed as Formula 5:

$${\bar{p}}_{i}=\lambda {\dot{p}}_{i}^{\left(unshared\right)}+\left(1-\lambda \right){\dot{p}}_{i}^{\left(shared\right)}$$ (5)

Where λ , $\lambda \in \left[0,1\right]$ , is the weighting parameter between the shared and non-shared fully connected layers.

### 3.3 Gait feature extraction

#### 3.3.1 Spatial features

The HoT framework is pruned along the temporal dimension, making it relatively insensitive to variations in specific joints. While GLA-GCN emphasizes the spatial relationships among joints, enabling fine-grained estimation of joint positions. The integration of HoT and GLA-GCN leverages their complementary strengths, enhancing prediction accuracy and improving the model’s generalization capability in the analysis of spatial parameters.

The computation of joint kinematics is facilitated through the implementation of Euclidean dot product formulation, enabling efficient derivation of inter-vector angular measurements.

The Knee Angle can be obtained according to Formula 6:

$$\theta_{knee\_i}=\cos^{-1}\left(\frac{\left(\vec{H_{i}K_{i}}\right)\cdot \left(\vec{A_{i}K_{i}}\right)}{\left\|\vec{H_{i}K_{i}}\right\|\cdot \left\|\vec{A_{i}K_{i}}\right\|}\right)$$ (6)

The Thigh Angle can be obtained according to Formula 7:

$$\theta_{thigh\_i}=\cos^{-1}\left(\frac{\left(\vec{H_{L\_i}K_{L\_i}}\right)\cdot \left(\vec{H_{R\_i}K_{R\_i}}\right)}{\left\|\vec{H_{L\_i}K_{L\_i}}\right\|\cdot \left\|\vec{H_{R\_i}K_{R\_i}}\right\|}\right)$$ (7)

Where $\theta_{knee\_i}$ , $\theta_{thigh\_i}$ denote the knee angle and thigh angle at frame i , respectively. The three-dimensional coordinates of the hip, knee, and ankle joints at frame i are represented as $H_{i}$ , $K_{i}$ , $A_{i}$ ; while $H_{L\_i}$ ¸ $H_{R\_i}$ , $K_{L\_i}$ and $K_{R\_i}$ represent the three-dimensional coordinates of the left and right hip and knee joints.

The corresponding joint angular velocity and angular acceleration video frame rate as Formulas 8, 9:

$$\omega_{i}=f\cdot \left(\theta_{i}-\theta_{i-1}\right)$$ (8)

$$a_{i}=f\cdot \left(\omega_{i}-\omega_{\left(i-1\right)}\right)$$ (9)

Where f represents the sampling frequency (30 Hz), $\theta_{i}$ is the joint angle at frame i , and $\omega_{i}$ is the joint angular velocity at frame i .

The step length for each frame is calculated as Formula 10:

$$L_{step\_i}=\left\|\vec{A_{Li}A_{Ri}}\right\|$$ (10)

Where $A_{Li},A_{Ri}$ denote the positions of the left and right ankle joints at frame i .

Based on the frame rate, the walking speed $v_{i}$ and walking acceleration $a_{step\_i}$ can be easily derived by Equations 11, 12:

$$v_{i}=f\cdot \left(L_{step\_i}-L_{step\_\left(i-1\right)}\right)$$ (11)

$$a_{i}=f\cdot \left(v_{i}-v_{\left(i-1\right)}\right)$$ (12)

The range of motion (ROM) is defined as Formula 13:

ROM=Max−Min (13)

##### 3.3.1.1 Temporal features

To extract time-related parameters, a multi-level feature extraction methodology is implemented. Based on the key frames extracted by HoT framework combined with initial 3D key point recognition, key frame filtering is performed on the GLA-GCN to reduce the overfitting of the GCN network.

**FIGURE 4:**
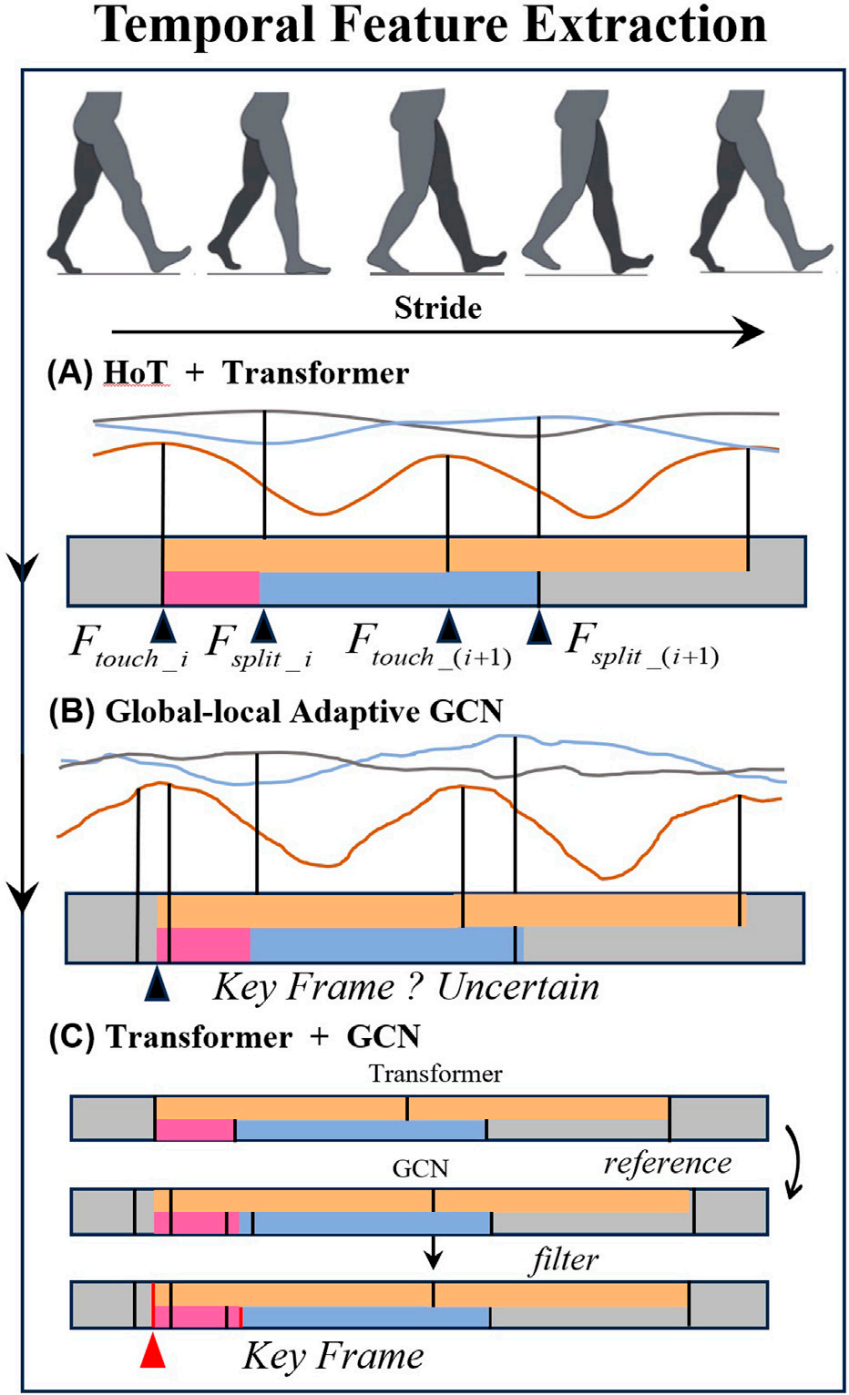
Temporal feature extraction diagram. **(A)** HoT + Transformer. **(B)** Global-local Adaptive GCN. **(C)** Transformer + GCN.

After obtaining the step length parameters, the corresponding step length time can be calculated using the keyframes associated with the step length and the frame rate. The maximum step length is recorded as the keyframe corresponding to the moment of heel strike, denoted as $F_{touch}$ . Additionally, the keyframe corresponding to the moment when the ankle joint reaches its maximum vertical height is recorded as $F_{split}$ , indicating the moment the toe leaves the ground, derived from the key points extracted using the Hot method. The keyframes extracted by the GLA method are filtered based on the reference of the keyframes obtained from the HoT method (Figure 4).

Finally, the various time-related parameters are calculated as Formulas 14–17:

$$t_{\left(single\_step\_time\right)\_i}=f\cdot \left(F_{touch\_\left(i+1\right)}-F_{touch\_i}\right)$$ (14)

$$t_{\left(single\_support\_time\right)\_i}=f\cdot \left(F_{split\_\left(i+1\right)}-F_{touch\_i}\right)$$ (15)

$$t_{\left(double\_support\_time\right)\_i}=f\cdot \left(F_{split\_i}-F_{touch\_i}\right)$$ (16)

$$t_{stride\_time}=f\cdot \left(F_{touch\_\left(i+2\right)}-F_{touch\_i}\right)$$ (17)

$t_{single\_step\_time},t_{single\_support\_time},t_{double\_support\_time}\text{ and }t_{stride\_time}$ represent the single step time, single support time, double support time, and stride time, respectively. For ease of calculation, $F_{touch\_i}$ denotes the keyframe at the moment of the ith heel strike, and $F_{split\_i}$ indicates the first keyframe following the ith heel strike when the toe leaves the ground.

The Double Proportion is defined as the ratio of double support time to single step time, as shown in Formulas 18, 19:

$$\begin{array}{c} Double\_proportion_{i}=t_{\left(double\_support\_time\right)\_i}/t_{\left(single\_step\_time\right)\_i}\cdot 100\% \end{array}$$ (18)

$$\begin{array}{c} Swing\_proportion_{i}=1-Double\_proportion_{i} \end{array}$$ (19)

The extracted temporal and spatial features are listed in Table 2.

**TABLE 2:** Summary of gait parameters.

Number	Feature name
	Temporal features
1	Single step time
2	Single support time
3	Double support time
4	Double proportion
5	Swing proportion
6	Stride time
	Spatial Features
7	Lknee angle ROM
8	Rknee angle ROM
9	Lknee angle velocity ROM
10	Rknee angle velocity ROM
11	Rknee angle acc. ROM
12	Lknee angle acc. ROM
13	Thigh angle max
14	Thigh angle velocity ROM
15	Thigh angle acc. ROM
16	Step length max

Note: Lknee represents left knee, Rknee represents right knee.

ROM, represents range of motion, acc. represents acceleration.

### 3.4 Statistical analysis

Gait feature parameters derived from video data in the experiment were assessed using the Intraclass Correlation Coefficient (ICC, 2 k) to evaluate reliability. The ICC calculation was based on Analysis of Variance (ANOVA), and following the threshold set by Koo and Li (Koo and Li, 2016) the ICC value greater than 0.75 is considered to indicate good consistency.

For validation experiments, temporal synchronization was established between video-captured heel-strike events and corresponding force-onset signatures recorded by the AMTI force platform, while simultaneously acquiring time-matched Vicon motion capture data. In the validation of spatial motion parameters, interpolation was used to match the frequencies of Vicon data (100 Hz) with video data (30 Hz).

The Pearson correlation coefficient (r) was employed to examine the degree of correlation between time-related parameters obtained from video data and those from the AMTI force platform, as well as spatial-related parameters obtained from Vicon. An r value of 0.7−0.9 indicates a high correlation, while a value of r≥0.9 suggests a very high correlation (Mukaka, 2012).

The Kruskal-Walli’s test was conducted to evaluate parameter differences among three subject groups: healthy controls, early Parkinson’s, and mid-stage Parkinson’s patients. Statistical significance was set at p <0.05 .

### 3.5 Classification of PD patients

A classification prediction model was developed to distinguish between healthy individuals and patients based on extracted temporal and spatial features. Considering the limited number of extracted features, Recursive Feature Elimination with Cross-Validation (RFECV) was employed to enhance the model’s generalization capability. Ten-fold cross-validation was employed to refine the temporal and spatial parameters extracted from the video data. To capture the complex relationships between features and enhance the model’s generalization ability, the random forest algorithm was employed as the base learner. Subsequently, RFECV was utilized to automatically identify the optimal feature subset for performance.

Using the final feature set, classification prediction was performed with machine learning methods including Adaptive Boosting (AdaBoost), Decision Tree (DT), K-Nearest Neighbors (KNN), Linear Discriminant Analysis (LDA), Logistic Regression (LOG), the Naive Bayes (NB), Random Forest (RF) and Support Vector Machine (SVM). Given the limited sample size in this study, ten-fold cross-validation and leave-one-out validation were employed to perform a comprehensive and fine-grained evaluation of the model’s performance. Performance metrics included accuracy (Acc), precision (Prec), recall (Rec), and F1 score. All analyses were conducted using Python.

## 4 Results

### 4.1 Correlation coefficients of three sets of video parameters

The features from the subject’s video data were extracted, and the ICC for three population feature groups was calculated. The results are shown in Table 3. The results indicate that *Single step time, Single support time, Double support time*, and *Stride time* exhibited excellent reliability and accurate estimation intervals in healthy individuals, early PD, and mid-stage PD, demonstrating strong intra-group consistency with statistically significant differences (ICC (2, k): (0.93, 0.93, 0.97); (0.94, 0.90, 0.91); (0.83, 0.86, 0.80); (0.95, 0.91, 0.91), *p* < 0.01). *Rknee angle acc. ROM, Lknee angle acc. ROM*, and *Step length max* also showed high consistency across groups, with significant inter-group differences (ICC (2, k): (0.90, 0.82, 0.89); (0.80, 0.83, 0.80); (0.87, 0.88, 0.89), *p* < 0.01).

**TABLE 3:** ICC results of video features of three groups of people.

Parameter	Healthy (25)			Early PD (19)			Mid PD (15)		
	ICC(2,k)	p	95%CI	ICC(2,k)	p	95%CI	ICC(2,k)	p	95%CI
Single step time	0.93	p < 0.01	[0.87,0.97]	0.93	p < 0.01	[0.84,0.97]	0.97	p < 0.01	[0.94,0.99]
Single support time	0.94	p < 0.01	[0.88,0.97]	0.90	p < 0.01	[0.79,0.96]	0.91	p < 0.01	[0.76,0.97]
Double support time	0.83	p < 0.01	[0.69,0.91]	0.86	p < 0.01	[0.70,0.94]	0.80	p < 0.01	[0.55,0.92]
Double proportion	0.70	p < 0.01	[0.36,0.84]	0.86	p < 0.01	[0.70,0.94]	0.72	p < 0.01	[0.40,0.89]
Swing proportion	0.70	p < 0.01	[0.36,0.84]	0.86	p < 0.01	[0.70,0.94]	0.72	p < 0.01	[0.40,0.89]
Stride time	0.95	p < 0.01	[0.89,0.98]	0.91	p < 0.01	[0.74,0.98]	0.91	p < 0.01	[0.70,0.98]
Lknee angle ROM	0.70	p < 0.01	[0.54,0.89]	0.74	p < 0.01	[0.46,0.89]	0.81	p < 0.01	[0.57,0.93]
Rknee angle ROM	0.75	p < 0.01	[0.51,0.88]	0.82	p < 0.01	[0.62,0.92]	0.75	p < 0.01	[0.41,0.91]
Lknee angle velocity ROM	0.83	p < 0.01	[0.67,0.92]	0.80	p < 0.01	[0.57,0.91]	0.77	p < 0.01	[0.44,0.92]
Rknee angle velocity ROM	0.78	p < 0.01	[0.57.0.89]	0.86	p < 0.01	[0.71,0.94]	0.84	p < 0.01	[0.62,0.94]
Rknee angle acc. ROM	0.90	p < 0.01	[0.80,0.95]	0.82	p < 0.01	[0.61,0.92]	0.89	p < 0.01	[0.73,0.96]
Lknee angle acc. ROM	0.80	p < 0.01	[0.62,0.91]	0.83	p < 0.01	[0.65,0.93]	0.80	p < 0.01	[0.51,0.93]
Thigh angle max	0.73	p < 0.01	[0.48,0.87]	0.85	p < 0.01	[0.68,0.93]	0.71	p < 0.01	[0.34,0.89]
Thigh angle velocity ROM	0.87	p < 0.01	[0.75,0.94]	0.67	p < 0.01	[0.31,0.86]	0.86	p < 0.01	[0.67,0.95]
Thigh angle acc. ROM	0.76	p < 0.01	[0.55,0.89]	0.69	p < 0.01	[0.35,0.87]	0.83	p < 0.01	[0.59,0.94]
Step length max	0.87	p < 0.01	[0.74.0.94]	0.88	p < 0.01	[0.74,0.95]	0.89	p < 0.01	[0.73.0.96]

Note: Lknee represents left knee, Rknee represents right knee, ROM, represents range of motion, acc. represents acceleration.

### 4.2 Correlation and difference between standard and monocular video

The parameters—*Single step time, Single support time, Double support time* (measured via the AMTI force platform), *Step length max, Lknee angle ROM,* and *Rknee angle ROM* (assessed using VICON)—were utilized as gold standards to evaluate the reliability and validity of parameters extracted from video. Correlations (*r*) and differences (*d*) between the gold standard and video-extracted features were calculated for both PD patients and healthy subjects, with results presented in Table 4.

**TABLE 4:** Correlation and difference between gold standard and video.

Parameter	Gold standard		Video data (transformer + GCN)					
	PD patients	Healthy	PD patients			Healthy		
	Mean (SD)	Mean (SD)	Mean (SD)	r	d	Mean (SD)	r	d
Single step time (s)	0.56 (0.06)	0.54 (0.08)	0.57 (0.06)	0.95	0.02	0.55 (0.09)	0.96	0.02
Single support time (s)	0.71 (0.09)	0.67 (0.12)	0.73 (0.09)	0.94	0.03	0.68 (0.11)	0.99	0.02
Double support time (s)	0.15 (0.04)	0.12 (0.04)	0.17 (0.04)	0.6	0.04	0.13 (0.04)	0.73	0.02
Step length max (mm)	491.3 (102)	553.5 (50.0)	441.3 (100.8)	0.88	53.7	456.9 (82.2)	0.92	63.2
Lknee angle ROM (rad)	0.82 (0.15)	0.91 (0.11)	0.78 (0.15)	0.85	0.18	0.74 (0.13)	0.9	0.13
Rknee angle ROM (rad)	0.88 (0.16)	0.89 (0.11)	0.76 (0.15)	0.8	0.18	0.74 (0.14)	0.88	0.13

Note: SD represents standard deviation.

The results in Table 4 indicate strong correlations between the gold standard and video-extracted parameters for *Single step time, Single support time, Step length max, Lknee angle ROM,* and *Rknee angle ROM* in both PD and healthy subjects (r = 0.95, 0.94, 0.88, 0.85, 0.80; r = 0.96, 0.99, 0.92, 0.90, 0.88). For time parameters, the differences in foot pressure and video-extracted data between the two groups are minimal (d = 0.02, 0.03, 0.04; d = 0.02, 0.02, 0.02). However, notable differences exist between the groups in VICON and video measurements (d = 53.7, 0.18, 0.18; d = 63.2, 0.13, 0.13).

### 4.3 Differences in video parameters among three types of people

**FIGURE 5:**
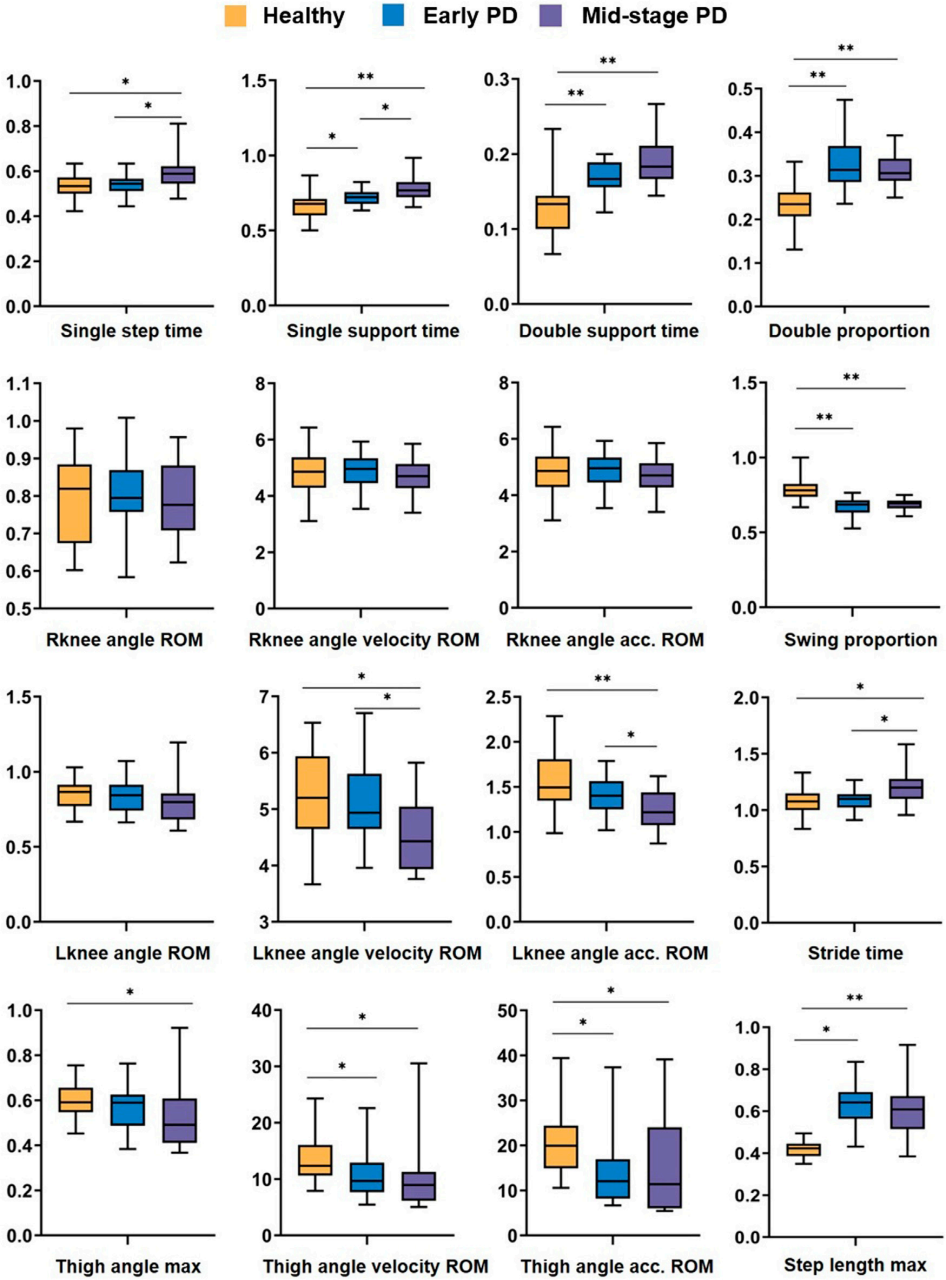
Analysis of difference results. Box plot of the difference analysis of temporal features and spatial features extracted from video data among healthy people (yellow), carly PD (bluc), and mid-stage PD (purple) groups, represents p-value < 0.05, ** represents p-value < 0.01.

Based on the Transformer and GCN methods, 6 temporal features and 10 spatial features of healthy people, early PD, and mid-stage PD in the video were extracted, the differences between the three groups were analyzed, and box plots were drawn. The results are shown in Figure 5. Among the 16 features, there were significant differences between healthy people and patients with mid-stage PD.

In terms of temporal features: there are significant differences (P < 0.05, P < 0.01, P < 0.01, P < 0.01) between healthy people and early PD patients in *Single support time*, *Double support time Double proportion*, and *Swing proportion*. There are significant differences in *Single step time* and *Stride time* between healthy and patients with mid-stage PD (P < 0.05, P < 0.05); in terms of spatial features: There are significant differences between healthy people and patients with mid-stage PD in *Lknee angle velocity ROM* and *Lknee angle acc. ROM* (p < 0.05, *p* < 0.01); there is no significant difference in the three characteristics of the right knee joint between the three groups of people; healthy and mid-stage PD have substantial differences in *step length max and the* 3 parameters of thigh joint angle characteristics; and there are differences between healthy people and early PD in *Thigh angle velocity ROM*, *Thigh angle acc. ROM, Step length max* (p < 0.05, p < 0.05, p < 0.05).

### 4.4 Health and patient classification results

The final feature set was obtained by using recursive feature elimination with cross-validation (RFECV). (*Single step time, Double support time, Double proportion, Stride time, Lknee angle ROM, Lknee angle velocity ROM, Rknee angle acc. ROM, Lknee angle acc. ROM, Thigh angle velocity ROM, Thigh angle acc. ROM, Step length max*), The above 11 features were input, and classification prediction was performed using machine learning methods such as AdaBoost, DT, KNN, LDA, LOG, NB, RF, and SVM. Ten-fold cross-validation was used to classify healthy people and PD patients. The accuracy (Acc), precision (Prec), recall (Rec), and F1 score tables are shown in Table 5. Except for the DT classifier, the accuracy, precision, recall, and F1 scores of all classifiers were higher than 90%, among which the RF classifier had the highest accuracy, precision, and F1 score (Acc = 93.33, Pre = 93.50, F1 = 94.07). The recall rates of LDA and LOG both reached 97.50.

**TABLE 5:** Classification results of PD patients and healthy elderly.

Model	Acc	Prec	Rec	F1
AdaBoost	90.00	91.50	94.17	91.53
DT	80.00	88.50	79.17	80.02
KNN	91.67	93.50	94.17	92.64
LDA	91.67	91.50	97.50	93.53
LOG	91.67	90.50	97.50	93.17
NB	91.67	93.00	94.17	92.60
RF	93.33	93.50	96.67	94.07
SVM	91.67	93.50	94.17	92.64

Note: Acc represents accuracy, Prec represents precision, Rec represents recall, F1 represents F1 score.

**FIGURE 6:**
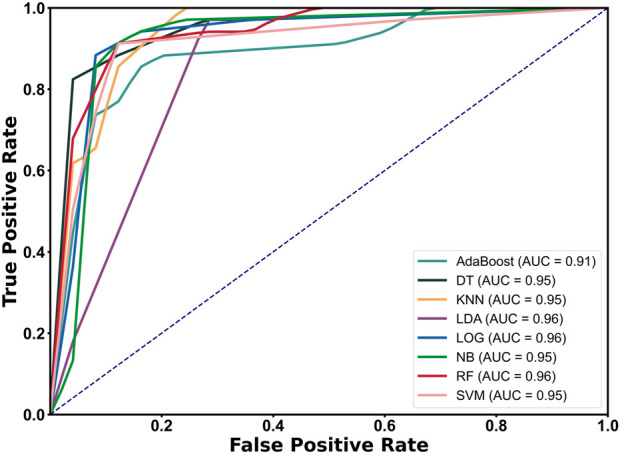
ROC curve and AUC calculation results of each classifier.

The Receiver Operating Characteristic (ROC) curve of the classifier algorithm was drawn and the Area Under the Curve (AUC) value was calculated using the leave-one-out validation method (11 features). The image is shown in Figure 6. The results show that the ROC curves all rise rapidly near the upper left corner, indicating that each separator has high sensitivity and specificity, and the overall performance of the classifier is excellent (AUC ≥0.91). Among them, the AUC of LDA, LOG, and RF all reached 0.96, reflecting their superior classification performance.

## 5 Discussion

This study aims to evaluate gait parameters in Parkinson’s disease (PD) patients using a markerless 3D human pose estimation method based on monocular video and to develop an accurate classification model for the early detection of PD. The main contributions include: 1) The proposed multi-level 3D pose estimation method based on Transformer and GCN can reliably and effectively provide the gait spatial and temporal parameters; 2) The established early prediction model for PD patients based on RF classifier can achieve a classification accuracy of 93.3%, demonstrating robust performance; 3) This work verifies the feasibility of the pose estimation method based on monocular video in patient motion capture, which is expected to become a new tool for clinical auxiliary diagnosis and rehabilitation evaluation.

Results 4.1 showed that the Intraclass Correlation Coefficient of multiple gait parameters of the subjects (healthy, early, and mid-stage PD patients) had ICC (2, k) > 0.7, indicating high repeatability. Armando et al. evaluated the repeatability of the Opal system in measuring the spatiotemporal gait characteristics of 45 PD patients. The results showed that most parameters had ICC <0.8, indicating low repeatability (Coccia et al., 2020). Liang et al. built a markerless pose estimation system based on OpenPose and 3DposeNet and evaluated the reliability and validity of the system for gait analysis in healthy elderly people. The ICC (C, 1) ranged from 0.538 to 0.765, which was relatively weaker than the present results (Liang et al., 2022). Hu et al. used a high-resolution graph convolutional multi-layer perception (HGcnMLP) 3D pose estimation framework based on monocular video to verify the reliability of spatiotemporal gait parameters such as joint angles in arthritis patients, and the ICC (2, k) ranged from 0.839 to 0.975, which was comparable to our results (Hu et al., 2024). Azhand et al. proposed a highly reliable gait parameter estimation algorithm based on monocular video, with the Intraclass Correlation Coefficient (ICC (2, k): 0.958 - 0.987) that was better than our results, but it was only verified in healthy adults (Azhand et al., 2021). In summary, the pose estimation method proposed in this study, which integrates Transformer and Graph Convolutional Network (GCN) architectures, demonstrates high repeatability.

Results 4.2 show the correlation analysis between the gait temporal parameters and spatial parameters estimated by the proposed method and the gold standard. Except for the *Double support time* (r = 0.60, 0.73), the correlation of all other parameters is *r* > 0.80, indicating high validity. Considering that the overall average *Double support time* is approximately 0.15 s, with the AMTI force plate (the gold standard) operating at 1,000 Hz and the video data captured at 30 Hz, the average error of 0.04 seconds—equivalent to roughly one frame (33 m)—falls within the acceptable range (Wu et al., 2022). Zachary et al. explored the comparison of gait parameters in PD patients based on markerless and marker motion capture, and the joint angle parameters showed excellent consistency in the sagittal plane (*r* > 0.90), but poor performance in the frontal and transverse planes (*r* < 0.60) (Ripic et al., 2023). Zhu et al. used a depth-based pose estimation algorithm to measure the joint angles of finger movements in real-time without contact, and compared them with the camera 3DMA gold standard system, showing excellent validity (0.88 < *r* < 0.97), which is comparable to our results (Zhu et al., 2021).

Results 4.3 showed significant differences in the most of gait parameters between different groups, indicating that there were significant differences between healthy elderly and PD patients (*p* < 0.05), and the differences in mid-stage PD were greater than those in early PD patients. However, no significant differences were observed among the three groups in the three characteristics of the right knee joint. In the video dataset, the probability of the left and right legs approaching the camera is approximately equal, minimizing the impact of one leg obstructing the other. This observation may be attributed to the fact that the right leg is the dominant leg for most individuals. Kiwon Park et al. compared the gait data of 10 healthy elderly individuals and 10 PD patients, reporting that PD patients exhibited significantly greater asymmetry across all lower limb joints (Park et al., 2016). Chen et al. compared the gait parameters of PD patients and healthy controls through differential analysis, thereby quantifying the severity of gait disorders and providing important information for the diagnosis and treatment of phenomena such as FOG (Chen et al., 2013). In summary, the analysis of correlation and differential results not only verifies the effectiveness of the proposed method but also provides a reference for the subsequent classification feature selection.

Results 4.4 shows the classification results of PD patients based on features extracted from monocular video and machine learning models, among which the RF classifier achieved an accuracy of 93.33%, showing good performance. Guo et al. proposed a novel two-stream spatiotemporal attention graph convolutional network (2s-ST-AGCN) for video assessment of gait movement disorders in PD patients, and the classification accuracy was 65.66% (Guo et al., 2022). Hu et al. used the monocular video HGcnMLP algorithm framework to well evaluate the balance ability of patients with musculoskeletal diseases, and the K-means++ clustering accuracy was 99% (Hu et al., 2024). Although better than our results, the healthy subjects were students aged 20–30 years, and there were significant gait differences between them and patients. Connie et al. used the AlphaPose pose gait analysis method to extract PD discriminative gait features from the original video, and the classification accuracy based on RF was 93%, which was comparable to our results. In addition, our work also made a detailed comparison of the gait differences between early and mid-stage PD patients (Connie et al., 2022). Katsuki et al. used a 2D video pose estimation algorithm to distinguish the feasibility of gait between PD and spinocerebellar degeneration, with accuracy, sensitivity, and specificity of 0.83, 0.88, and 0.78 respectively (Eguchi et al., 2024). The above results show that the 3D pose estimation method has better performance than 2D in gait evaluation. In summary, this study preliminarily verified the feasibility of the monocular video estimation method for clinical diagnosis and gait evaluation of PD patients.

We acknowledge some limitations of this work. First, during the experimental data collection, to use the ATMI plantar pressure data as the standard for time parameters, there were only 2 complete gait cycles per video on average that could be reliably identified and verified. Next, the experimental settings and data collection schemes should be improved to provide more gait information; second, the proposed pose estimation method provides only a limited set of gait temporal and spatial parameters, and it needs to estimate additional parameters that reflect external manifestations of Parkinson’s disease (PD), such as the center of mass and gait variability. Moreover, it does not extract advanced features such as gait symmetry or joint coupling. Based on the differences in parameters related to the left and right joints observed in Result 4.3, future research could further explore and validate the differences in corresponding parameters between the dominant and non-dominant legs across different groups. This could provide valuable insights for developing more efficient and effective early screening methods for PD in clinical practice; finally, the established classification model only considers the binary classification situation and a disease severity evaluation model for PD patients can be established in the future. Nevertheless, this work is an effective attempt to use markerless pose estimation methods in PD gait monitoring and evaluation, providing a new solution for early intervention of PD patients.

## 6 Conclusion

The verification results of gait spatial parameters and temporal parameters in this study show that the proposed markerless 3D pose estimation method has good reliability and effectiveness, and the superiority of the classification results shows that the constructed prediction model can be used for early monitoring of PD patients. Due to the advantages of simplicity, portability, cheapness, and easy operation, the proposed method is expected to provide a clinical alternative to human motion capture in the future and provide a new and easy-to-operate tool for remote gait monitoring and functional evaluation of PD patients.

## Data Availability

The original contributions presented in the study are included in the article/supplementary material, further inquiries can be directed to the corresponding authors.
